# The Fibrinolytic System of the Chacma Baboon (*Papio ursinus*): Implications for Non-Human Primate Haemostatic Disease Models

**DOI:** 10.3390/ijms27219947

**Published:** 2026-11-06

**Authors:** Jaco Joubert, Muriel Meiring, Walter Janse Van Rensburg

**Affiliations:** 1 Department of Haematology and Cell Biology, School of Pathology, Faculty of Health Sciences, University of the Free State, Bloemfontein 9301, South Africa; joubertj@ufs.ac.za; 2 National Health Laboratory Service, Universitas Academic Laboratories, Haematology, Bloemfontein 9301, South Africa; 3 Human Molecular Biology Unit, School of Biomedical Sciences, Faculty of Health Sciences, University of the Free State, Bloemfontein 9301, South Africa; jansevrwj@ufs.ac.za

**Keywords:** fibrinolysis, baboon model, Alteplase, Streptokinase, plasmin generation potential, *Papio ursinus*

## Abstract

Baboon models are useful for investigating haemostatic disorders and potential novel treatments. The fibrinolytic system is an integral component of these models and an important potential therapeutic target. We investigated the fibrinolytic system of the Chacma baboon (*Papio ursinus*), including the effects of sex and ABO blood group. Tissue-type plasminogen activator (tPA), plasminogen activator inhibitor-1 (PAI-1), plasminogen, plasmin-α2-antiplasmin (PAP) complexes, thrombin activatable fibrinolysis inhibitor (TAFI), α2-antiplasmin, fibrinogen levels, and clot lysis times of 40 adult Chacma baboons were assessed. Plasminogen activation using escalating concentrations of streptokinase vs. alteplase was determined and compared with normal human plasma. The results were compared between O and non-O ABO blood groups, and between males and females. PAP complexes and fibrinogen were mostly within the human reference interval, while tPA, PAI-1 and α2-antiplasmin tended towards the lower limit. Plasminogen and TAFI were virtually undetectable with the human ELISA kits used. Alteplase was a potent plasminogen activator compared with streptokinase (*p* < 0.0001), resulting in brisker clot lysis in baboon than in human plasma (10.8 vs. 13.4 min). Blood group O baboons had higher fibrinogen levels (*p* = 0.0355), while males had lower fibrinogen (*p* = 0.0134) but higher PAP complex concentrations (*p* = 0.0188). Except for plasminogen and TAFI, the Chacma baboon fibrinolytic system appears broadly similar to its human counterpart. Streptokinase does not meaningfully activate Chacma baboon fibrinolysis, but alteplase elicits greater plasminogen activation than in humans. These findings, as well as sex- and ABO-blood-group-related differences in fibrinogen, should be considered when designing future pre-clinical drug testing experiments in this species.

## 1. Introduction

Chacma baboon (*Papio ursinus*) models of human diseases are useful for establishing a proof-of-concept of novel treatment approaches and are commonly used as non-human primate models of human haemostatic disorders. However, surprisingly little is known about their fibrinolytic systems. Evaluations of certain compounds (such as recombinant glycosylated human interleukin-6, both in normal baboons [1] and in a disseminated intravascular coagulation (DIC) model [2], soluble-fibre concentrate in obese baboons [3], and puff adder venom [4] in normal baboons) have included assessments of their effects on *Papio ursinus* fibrinolysis, with varying depth and levels of sophistication. However, perhaps apart from a 1959 study which assessed the effects of the menstrual cycle on the euglobulin clot lysis time in three baboons [5], and a 2012 study which assessed similarities in thromboelastometric findings between humans and baboons [6], there are no publications focusing specifically on the *Papio ursinus* fibrinolytic system in general, its level of homology with human fibrinolysis, or its interplay with other components of the haemostatic system, under physiological conditions. The reason for this is unclear, but it may simply be a consequence of the high priority awarded to other haemostatic components (such as platelets, ADAMTS13 and Von Willebrand Factor) in previous *Papio ursinus* animal model research [7], which may have come at the expense of studies focusing purely on fibrinolysis.

Notably, although the in vivo effects of various systemic thrombolytic drugs such as recombinant single-chain urokinase-type plasminogen activator (uPA), recombinant tissue-type plasminogen activator (tPA), and a hybrid recombinant plasminogen activator consisting of an anti-fibrin antibody combined with low-molecular-weight single-chain uPA, have been investigated previously in an unspecified baboon species [8], prior to our own research efforts [9], only two studies had investigated the in vivo responses of the *Papio ursinus* fibrinolytic system to systemic thrombolytic drugs: Van Zyl et al. investigated the utility of the chimeric protein PLATSAK (a recombinantly produced antithrombin-antiplatelet-staphylokinase conjugate) in a *Papio ursinus* arterial and venous thrombosis model [10], and Franz et al. investigated the effect of tPA [11] as part of a more extensive study assessing the utility of rotational thromboelastography in the quantification of the resultant lytic state during thrombolytic therapy [12].

Although there is a dearth of practical, published experience with the thrombolytic drugs (especially streptokinase) in *Papio ursinus* in vivo, other baboon models (most notably *Papio hamadryas*) have been used successfully to investigate the effects of many of the thrombolytic drugs (including streptokinase). Most of these studies were performed during the pre-clinical development phases of these drugs for the treatment of thrombotic conditions such as acute myocardial infarction.

In 1966, Hampton and Matthews studied the in vitro effect of streptokinase on fibrinolysis in the plasma of an unnamed baboon species. They found that although high-dose urokinase was able to activate baboon fibrinolysis to an extent comparable with humans, streptokinase was not [13].

Collen et al. investigated the comparative thrombolytic properties and immunogenicity of streptokinase and the (at the time) relatively poorly studied thrombolytic drug recombinant staphylokinase in a *Papio hamadryas* arterial and venous thrombosis model [14]. Streptokinase was found to be relatively inefficient at dissolving platelet-rich thrombi in their femoral arterial eversion graft model, and this was not due to anti-streptokinase antibodies or other inhibitors. The shortest time to reflow was over an hour (63 ± 33 min), and persistent patency was only achieved in one of the twelve test animals, at a dose of 0.50 mg/kg. In comparison, staphylokinase resulted in reflow in 24 ± 12 min at a dose of 0.25 mg/kg, and persistent patency was achieved in four of the twelve test baboons.

However, systemic infusion of streptokinase led to progressive, dose-dependent clot lysis in baboons with jugular vein thrombosis (comparable to the lysis obtained with staphylokinase), indicating meaningful fibrinolytic activation and that the relative potencies of these two drugs for activation of the *Papio hamadryas* fibrinolytic system in vivo may be comparable. The authors caution that in vitro clot lysis systems may not be representative of in vivo thrombolytic performance and that results obtained in different animal models cannot always be extrapolated to human clinical conditions [14].

Different experimental animal models also cannot be extrapolated to one another. Variability in the reactivity of human, rabbit and hamster [15], as well as human, equine, feline, canine, bovine, and leporine [16], fibrinolytic systems have been reported for streptokinase and were also investigated *inter alia* in *Papio hamadryas* baboons by Lijnen et al. [17]. In their study, streptokinase was a relatively poor plasminogen activator in dogs, rabbits, hamsters, rats and *Papio hamadryas* when compared to humans, with significant interspecies variation.

Collen et al. investigated this interspecies variability in the human, baboon, leporine, canine, and bovine fibrinolytic systems to streptokinase and recombinant staphylokinase activation [18]. They concluded that this variability is determined by the ability of these compounds to form complexes with plasminogen, by the catalytic efficiencies of the resultant complexes for the activation of autologous plasminogen, and by the rate at which these complexes are inhibited by α_2_-antiplasmin [18]. Notably, the plasminogen molecule of the (unspecified) baboon species tested in their experiment was unable to form stable stoichiometric complexes with streptokinase, but did react stoichiometrically and quantitatively with staphylokinase, although these complexes had low catalytic efficiencies.

Since there are demonstrable differences between the propensities of the human, *Papio hamadryas,* and other unnamed baboon fibrinolytic systems for activation by streptokinase, it is conceivable that there may also be differences across the various *Papio* spp.

As part of a larger research effort investigating the pathogenesis and treatment of acute acquired Thrombotic Thrombocytopenic Purpura (aTTP) in humans, we studied the in vitro and in vivo effects of low-, intermediate-, and high-dose streptokinase in the established Chacma baboon model of aTTP [9]. This study was performed to ascertain whether a more extensive-scale baboon study of streptokinase would be feasible, but demonstrated that streptokinase was not able to resolve aTTP in the Chacma baboon aTTP model, possibly due to its limited activation of the *Papio ursinus* fibrinolytic system, in contrast to its potent effects in humans [17].

Ultimately, this study exposed significant gaps in our knowledge of Chacma baboon fibrinolysis. Clearly, a more detailed haematological characterisation of the Chacma baboon’s fibrinolytic system was required, since it is commonly used as a model of several human haemostatic diseases, where the role of fibrinolysis is important.

We now aimed to determine provisional normal reference intervals in the Chacma baboon for the most central components of the fibrinolytic system, namely tPA, plasminogen activator inhibitor-1 (PAI-1), plasminogen, plasmin-α2-antiplasmin (PAP) complexes, thrombin activatable fibrinolysis inhibitor (TAFI), α2-antiplasmin, and fibrinogen, and compare them with known human data and other animal model data, where available/applicable. In addition, we aimed to evaluate the Chacma baboon’s overall in vitro clot lysis ability by determining modified clot lysis times and by determining and comparing the maximum in vitro plasmin activity generated in *Papio ursinus* plasma when activated with streptokinase vs. recombinant human tPA (alteplase). As a secondary objective, we also investigated whether any of the parameters outlined above are influenced by sex or ABO blood group in the Chacma baboon.

## 2. Results

The statistical analyses of the fibrinogen level and the various components of the fibrinolytic system are summarised in Table 1 below. The fibrinogen levels were generally consistent with human values, with nine baboons (22.5%) falling below the human reference range cut-off value of 1.7 g/L. Plasminogen levels were virtually undetectable with the human ELISA kit used, with all values measuring far below the lower human reference range cut-off value for this kit of 60 μg/mL, even after the assay had been repeated on a second set of aliquots. tPA levels tended to the lower limit of the human reference range, with 21 baboons (52.5%) below the human reference range cut-off. The tPA central 95% interval was much narrower (1.4 ng/mL) than the range expected in humans (6 ng/mL). PAI-1 levels also tended towards the lower limit of the human reference range, and 11 baboons (27.5%) had values below the cut-off value. All animals had PAP complex levels within the human reference range, but unlike what may be seen in humans, no baboon had a PAP complex level of 0 ng/mL. TAFI was not detectable in 15 baboons (37.5%), with the rest of the values obtained also far below the human reference interval for this kit. Note that the Chacma baboon TAFI results are reported in ng/mL in Table 1, but that human reference intervals for this analyte (including the reference interval supplied by the manufacturer of the ELISA kit used in this experiment) are usually in the μg/mL range.

The very low, virtually undetectable levels of plasminogen and TAFI suggest that these two kits may not be suitable for quantifying these proteins in *Papio ursinus* plasma. Moreover, this suggests fundamental structural differences between these two proteins and their human equivalents, since ELISA kits designed for use in humans could not generate results with *Papio ursinus* plasma. This is likely due to structural differences that affect the binding of the antibodies used in these kits. However, the inability of a human-directed ELISA to detect a baboon protein does not by itself establish a specific structural difference in that protein, and this finding is ultimately hypothesis-generating.

α2-antiplasmin concentrations varied dramatically between individual animals with a CV of 270.5%. Although α2-antiplasmin concentrations also tended to be well below the human reference interval for this kit (25–50 μg/mL), five animals (12.5%) did have values >10 μg/mL, with a value as high as 95.8 μg/mL obtained in one animal. In contrast to the plasminogen and TAFI data, where these analytes were virtually undetectable in all animals assayed, the α2-antiplasmin results suggest that this particular kit is suitable for use in *Papio ursinus* (although the kit does seem to be reasonably species-specific, with <3% reactivity for monkey, mouse, rat, or cow α2-antiplasmin according to the manufacturer), and that Chacma baboon α2-antiplasmin concentrations are indeed lower than human concentrations.

All baboon data had a normal distribution, confirmed with either a Shapiro–Wilk or D’Agostino-Pearson normality test.

**ABO blood group results and influence on assay parameters**

Most animals (*n* = 30; 75%) were ABO blood group O, while the remainder (*n* = 10; 25%) typed as subgroups of A. No baboons typed as group B or AB. There were no significant differences (*p* < 0.05) found between the different blood groups for any of the fibrinolytic system parameters measured. However, baboons with blood group O had higher fibrinogen levels (*p* = 0.0355, Hedges’ g = 0.7914, 95% CI: 0.0689–1.514) than baboons typed as subgroups of A.

**Influence of animals’ sex**

Most animals were female (*n* = 26; 65%, males: *n* = 14; 35%). Males had lower fibrinogen levels (*p* = 0.0134, Hedges’ g = 0.7522, 95% CI: 0.0944–1.4100) than females, but higher PAP complex concentrations (*p* = 0.0188, Hedges’ g = 0.8252, 95% CI: 0.1632–1.4872). Animals’ sex did not influence any of the other parameters assayed.

**Plasmin activity**

Plasmin activity was assessed after induction with the thrombolytic drugs streptokinase and tPA at baseline and at escalating concentrations. The baseline and thrombolytic drug-induced activities obtained during the concentration escalation studies are presented separately below. Unless otherwise stipulated, differences between activators were assessed using paired Student’s *t*-tests.

**Baseline plasmin activity**

**Figure 1:**
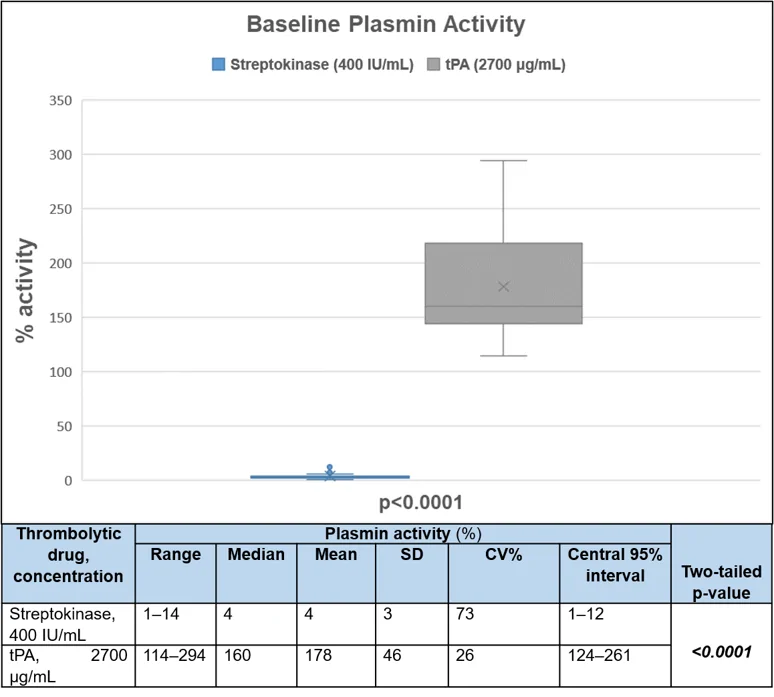
Box-and-Whisker plot of baseline plasmin activity, expressed as a percentage of the activity induced in pooled normal human plasma, using streptokinase and tPA (tissue-type Plasminogen Activator) as activators (*n* = 40).

The baseline plasmin activities (defined as the activity obtained with the addition of activator at concentrations representing approximately 0.03% of the total STEMI treatment dose per millilitre in humans—please see Materials and Methods) induced by streptokinase and tPA are compared in Figure 1 below. Compared with tPA, very little activity could be demonstrated with streptokinase as an activator (*p* < 0.0001). tPA was able to induce plasmin activity in *Papio ursinus* plasma to a greater extent than in pooled normal human plasma, with the activity in all baboon samples exceeding 100% of human activity.

**Thrombolytic drug-induced plasmin activity—concentration escalation studies**

The plasminogen activation potentials of streptokinase and tPA, using escalating concentrations of activator, are shown in the figures below as percentages of the activities obtained in pooled normal human plasma, using the baseline concentrations defined above, i.e., 400 IU/mL for streptokinase and 2700 μg/mL for tPA.

**Streptokinase plasminogen activation potential**

**Figure 2:**
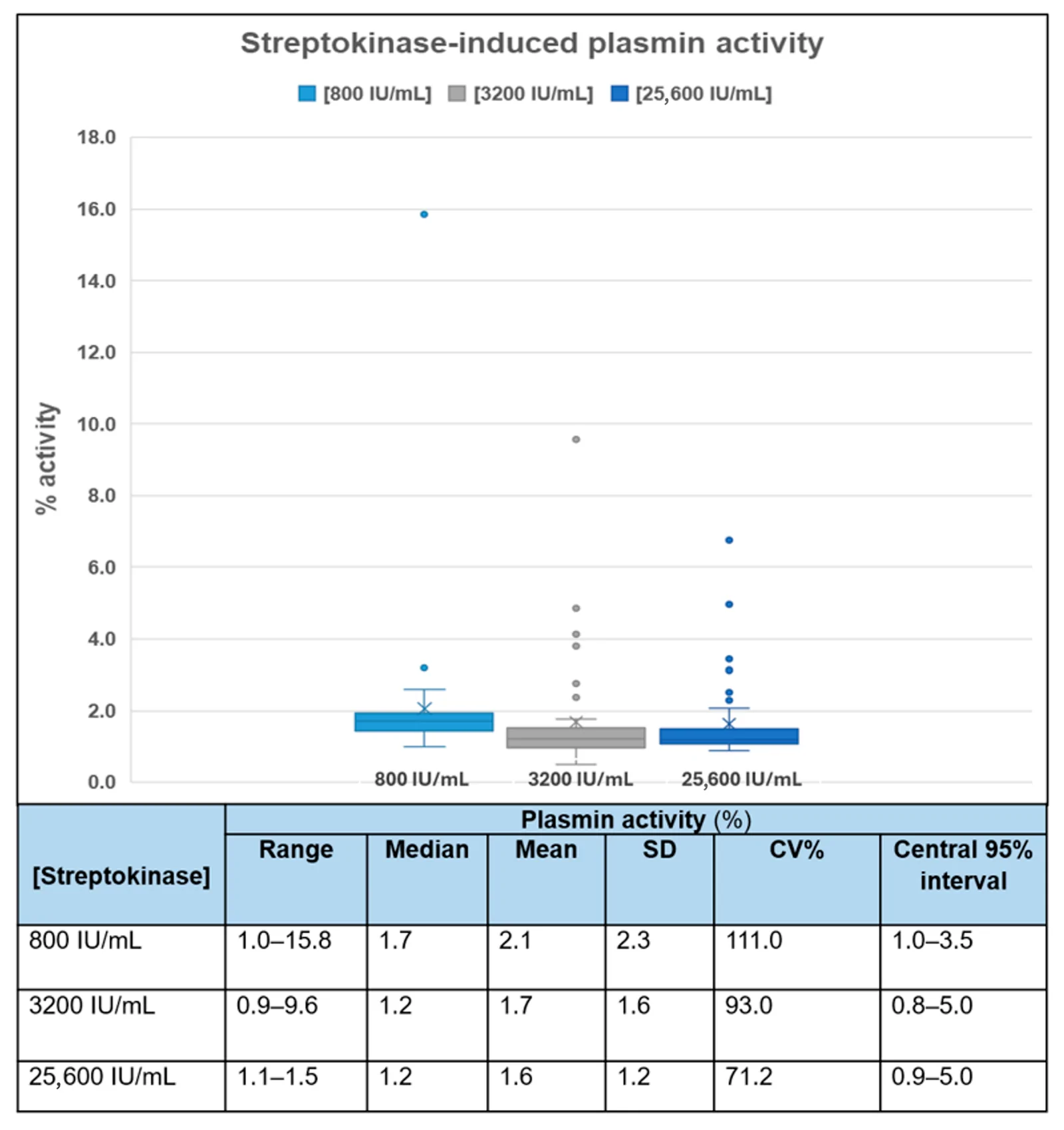
Box-and-Whisker plot of plasmin activity elicited by increasing concentrations of streptokinase, expressed as a percentage of the baseline activity induced in pooled normal human plasma (*n* = 40).

The plasmin activities obtained with escalating streptokinase concentrations are shown in Figure 2 below. Virtually no activity could be demonstrated at any concentration, even when the streptokinase stock solution [300,000 IU/mL] was used neat on a pooled Chacma baboon plasma sample for confirmation. A maximum concentration was thus not identified.

**tPA plasminogen activation potential**

**Figure 3:**
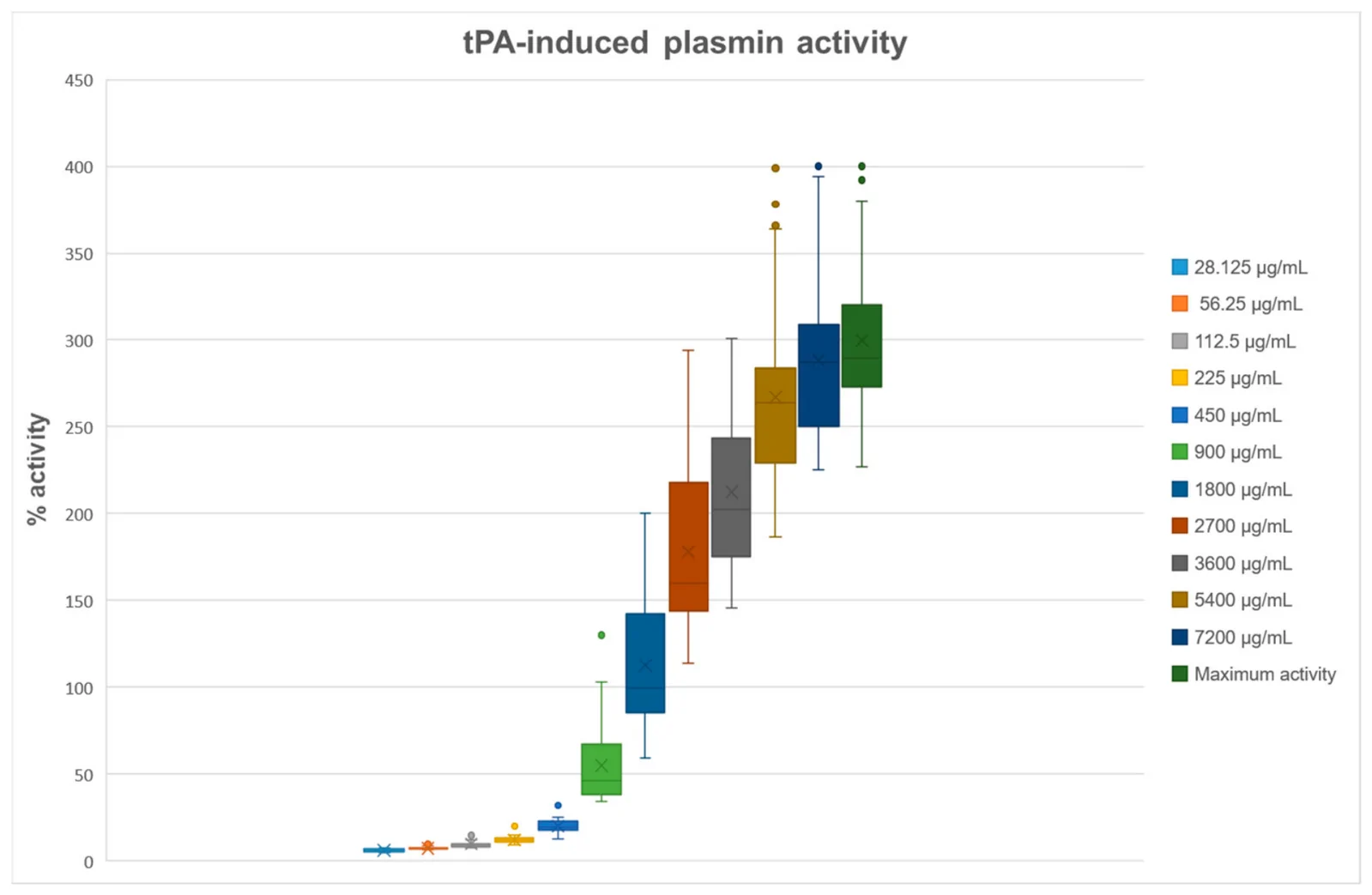
Box-and-whisker plot of plasmin activity elicited by increasing concentrations of tPA, expressed as a percentage of the baseline activity induced in pooled normal human plasma (*n* = 40).

Figure 3 below demonstrates the concentration-dependent increases in plasmin activity encountered when Chacma baboon plasma samples were exposed to escalating concentrations of tPA. Plateaus in activity were encountered in all samples at concentrations of either 5400 μg/mL (*n* = 9) or 7200 μg/mL (*n* = 31).

**Modified clot lysis time**

All baboon samples (Range: 7.9–13.2 min; Median: 10.2 min; Mean: 10.4 min; SD: 1.2; CV%: 11.6; Central 95% interval: 8.5–12.6 min; Pooled baboon sample: 10.8 min) had modified clot lysis times less than the pooled normal human plasma sample (13.4 min), indicating relatively brisk in vitro clot lysis when activated by tPA at a concentration of 225 ng/mL, which was the lowest concentration at which complete clot lysis could be elicited in both baboon and human plasma. A modified clot lysis assay using streptokinase as activator was not attempted, based on the findings above. A normal human reference range is not available for this modified assay. No lysis was observed in either of the two negative controls (pooled baboon plasma sample and pooled normal human plasma sample, with no added tPA).

## 3. Discussion

**Fibrinogen—the potential size of the fibrinolysis substrate pool**

The fibrinogen concentration range was generally comparable to the human range, albeit with lower cut-off values at both ends of the provisional reference interval. This contrasts with the findings of Schöchl et al. that fibrinogen levels are significantly lower in Chacma baboons than in humans [6]. They report a median fibrinogen concentration of 1.026 g/L (*n* = 25, interquartile range: 0.785–1.236 g/L), which is 44% lower than our median of 1.83 g/L, obtained using the same (Clauss) method. It is also well below the human reference range for our assay (1.7–4.2 g/L). Although their study only included male baboons, which may explain this discrepancy, the 14 male baboons included in our study still had a median fibrinogen concentration of 1.67 g/L, which is only 9% lower than the overall median. However, male baboons in our study did have significantly lower fibrinogen levels than the female animals (*p* = 0.0134, Hedges’ g = 0.7522, 95% CI: 0.0944–1.4100).

Data from the aTTP Chacma baboon model are limited to the baseline fibrinogen levels from our in vivo streptokinase study [9] and the initial description of the model [26]. In the other preclinical studies, baseline fibrinogen concentrations were either not assessed [27,28] or assessed but not reported [29]. Although only male baboons (*n* = 6) were used for the initial description of the model, Feys et al. report a mean fibrinogen level of 3.0 g/L (SD = 0.5 g/L), which is comparable to human ranges, using the same (Clauss) method as the present study [26].

Although fibrinogen levels in this species are therefore generally comparable to human ranges, viewed as a whole, these studies suggest that interindividual variation may be more considerable in Chacma baboons than previously anticipated, which should be considered when designing future experiments in this species. Ultimately, there does appear to be an acceptable level of homology between Chacma baboons and human fibrinogen so that translatability of results is retained.

**The fibrinolytic system and its potential therapeutic manipulation with thrombolytic drugs**

**tPA and its principal inhibitor, PAI-1**

Like the fibrinogen data, tPA and PAI-1 levels were generally comparable to human ranges but also with lower cut-offs at both ends of the provisional reference interval. Although one may speculate whether this reflects truly lower levels in *Papio ursinus* plasma or is also merely a reflection of less than perfect cross-reactivity of baboon proteins with human assays, it does also confirm at least some homology with the human fibrinolytic system, of at least one plasminogen activator and its inhibitor. This has important implications for the use of thrombolytic drugs in the aTTP model. Firstly, since Chacma baboon tPA was found to be quantitatively similar to human tPA, and (since the assay was able to generate results) possibly structurally similar as well, the use of human tPA in this model may have clinical effects very similar to its use in humans—a hypothesis worth exploring in vivo. Secondly, and perhaps more importantly, with similar PAI-1 levels (and possibly structure) to humans, similar levels of inhibition of any exogenously administered tPA can be expected.

Prior to our present study, tPA levels had not been specifically assessed in normal Chacma baboons, although tPA levels were measured by Franz et al., as part of their investigation of rotational thromboelastography as a tool to quantify the ensuing ‘lytic state’ during thrombolytic therapy with tPA in this species [11,12]. Baseline tPA levels in their study (approximate mean = 16 ng/mL) are higher than our levels (mean = 2.0 ng/mL, range: 1.4–4.0 ng/mL), again reiterating the value of establishing exploratory reference ranges in this species for contextualization of results.

Although one study, conducted in an Olive baboon (*Papio anubis*) sepsis model, reports baseline tPA concentrations of approximately 0 ng/mL [30], most preclinical studies in other baboon species report higher levels than found in our present study. Higher baseline tPA levels are reported in a *Papio hamadryas* heatstroke model study of the effects of recombinant activated Protein C on endothelial injury and microparticle formation, for instance (approximate mean = 15 ng/mL, *n* = 14) [31], as well as in a study of the effects of hypovolaemia (induced by bleeding vs. lower body negative pressure) on an unnamed baboon species (approximate mean = 25 ng/mL, *n* = 13), which, interestingly, also revealed a subset of baboons with baseline hyperfibrinolysis, found to be strongly associated with tPA levels [32]. In this study, perhaps counterintuitively, hyperfibrinolytic baboons (defined using thromboelastography) had the lowest tPA levels at baseline, although the authors concede that correlation does not imply causation [32]. Whether these findings can be directly extrapolated to the Chacma baboon is open to speculation. The possibility of the existence of a hyperfibrinolytic subset of baboons (which can possibly be predicted based on tPA levels) is intriguing, however, and worth exploring (perhaps also using thromboelastography) in the Chacma baboon, as this has definite implications for the use of thrombolytic drugs in the aTTP model.

Regardless, although tPA levels may be physiologically lower in the Chacma baboon than in other baboon species, the mean Chacma baboon tPA concentrations obtained in our study did fall within the human reference range. In contrast, the approximate mean baseline concentrations reported in other baboon species [30,31,32] do not, perhaps suggesting a higher level of homology of Chacma baboons to humans. This is encouraging in terms of the aTTP model’s translatability and future use to investigate the potential therapeutic effects of thrombolytic drugs. Moreover, the baseline approximate mean PAI-1 concentration of 2 ng/mL (*n* = 13) reported by Zaar et al. [32] is below the human reference range of 7–43 ng/mL. Our mean of 11.3 ng/mL (range: 1.9–32.3 ng/mL; *n* = 40) was within the human reference range, suggesting good comparability of Chacma baboon and human PAI-1 physiology. Therefore, the main inhibitor of any exogenously administered tPA appears to possibly have more similar levels to humans in the Chacma baboon than in other baboon species, reiterating the suitability of the Chacma baboon over other baboon species to preclinically investigate the in vivo effects of thrombolytic drugs in aTTP.

In addition, both tPA and PAI-1 have been studied as potential biomarkers for the early diagnosis of burn-induced coagulopathy, for example [33], and should their utility be explored in a *Papio ursinus* model, our exploratory data may be useful to provide context.

**PAP complexes—an indication of background plasminogen activation**

PAP complex concentrations were comparable to human levels, although, unlike what may be seen in humans, no animal had a PAP complex level of 0 ng/mL (the lowest value obtained was 99.9 ng/mL), possibly suggesting higher background plasminogen activation in Chacma baboons than in humans. It is consistent with the approximate mean baseline values of 30–40 ng/mL reported by Zaar et al. [32] for an unspecified baboon species, but not with the mean baseline concentrations of approximately 0 ng/mL reported by Keshari et al. [30] in a study involving Olive baboons (*Papio anubis*), suggesting possible interspecies differences in background plasmin activity, even across different baboon species. However, because PAP complexes are cleared approximately five times faster than α_2_-antiplasmin [34] or plasminogen [35], and concentrations may, therefore, decrease within minutes [36], the PAP complex levels essentially provide a snapshot of current, ongoing plasminogen activation, so that non-physiological, transient plasminogen activation (related for example to the sedation and handling of the animals) cannot be excluded as a reason for the apparently higher background plasminogen activation evident from our data. Nevertheless, our data ultimately confirm that Chacma baboon PAP complex levels (and therefore background plasminogen activation) are generally comparable to human levels, reinforcing the suitability of the Chacma baboon for preclinical studies involving fibrinolysis.

PAP complex levels were only assessed in our in vivo streptokinase pilot study [9] and not in any of the other preclinical studies in the Chacma baboon aTTP model [26,27,28,29]. In our pilot study, the low- and intermediate-dose test animals had a baseline PAP complex level (112 ng/mL) within the provisional reference interval now derived from our current data (109.0–215.8 ng/mL), but the high-dose test animal did not. A baseline PAP complex level of 390 ng/mL suggests that this baboon was possibly already afflicted with either a thrombus or some other illness, since our current data would indicate that this animal demonstrated supra-physiological plasminogen activation already at baseline. Again, these observations demonstrate the value of credible reference intervals in this model for the common fibrinolytic system parameters. Confirmation of the exploratory reference ranges generated by our data will now be desirable, preferably in an independent, larger cohort.

**Plasminogen—a dissimilar driver of fibrinolysis in the Chacma baboon?**

Plasminogen was virtually undetectable using the Technozym^®^ Glu-Plasminogen ELISA kit (Technoclone GmbH, Vienna, Austria), with the central 95% interval (1.8–4.2 μg/mL) far below the human reference range of 60–250 μg/mL for this kit [20] despite repeating the assay on an alternative set of aliquots. It is unlikely that this reflects truly lower levels in the Chacma baboon since the discrepancy is so large. It is more plausible that the selected ELISA kit may not be suitable for quantifying this protein in Chacma baboon plasma since it is designed for use in humans. Importantly, this finding suggests possible fundamental structural differences between this protein and its human equivalent, although it must be noted that the inability of a human-directed ELISA to detect another primate protein does not necessarily, by itself, confirm a specific structural difference in that protein. However, several lines of evidence from our study support the hypothesis that Chacma baboon plasminogen is not only structurally different from human plasminogen but functionally dissimilar as well.

Although ultimately speculative, our plasminogen concentration data indicate that either the monoclonal murine anti-human plasminogen capture antibodies or the POX-conjugated monoclonal murine anti-human plasminogen detection antibodies (or both) used in the Technozym^®^ ELISA kit did not bind to Chacma baboon plasminogen, implying a structural difference in at least one domain. Our observations that streptokinase cannot induce any meaningful plasmin activity in Chacma baboon plasma, whereas comparable amounts of tPA led to greater plasmin activity (as well as a more remarkable clot lysis ability) in Chacma baboon plasma than found in humans, suggest significant functional differences.

However, although plasminogen levels were not assessed in the other preclinical studies conducted in the Chacma baboon aTTP model [26,27,28,29], our pilot study [9] revealed baseline plasminogen levels that were either within (180 μg/mL, in the low & intermediate-dose test animal) the human reference range (60–250 μg/mL) or drastically above it (2562 μg/mL, in the high-dose test animal) using an ELISA kit from the same manufacturer (Technoclone GmbH, Vienna, Austria). This also raises the possibility that preanalytical factors may have influenced the current results. Since, under certain circumstances (such as during thrombolytic therapy or with endogenous hyperfibrinolysis), Glu-plasminogen may be partially degraded after blood collection [20], and since plasminogen can also be completely degraded by fairly ubiquitous proteinases such as elastase, procathepsin D, or metalloelastase [37], it is conceivable that Chacma baboon plasminogen may be more sensitive to post-collection degradation than previously anticipated. However, the preanalytical conditions of the present study were identical to those of the pilot study.

Although any speculation on possible differences in individual domains or residues is ultimately merely hypothesis-generating, it would be interesting to investigate the level of plasminogen homology in the Chacma baboon, Olive baboon, and man by sequencing this gene in the Chacma baboon in a future study, especially in light of the lack of streptokinase-induced plasmin activity encountered in our present study. Additional plasminogen functional testing to supplement our ELISA-generated plasminogen concentration data would also be useful.

**TAFI—a dissimilar indirect inhibitor of fibrinolysis in the Chacma baboon?**

Since published mean concentrations for TAFI in various human populations range from 5.71 μg/mL [38] to 9.6 μg/mL [39], and since TAFI is generally deemed to have a human reference interval of 4–15 µg/mL (70–275 nmol/L) [40,41,42], it is clear that virtually no TAFI was detected in our baboon plasma samples, even with the high sensitivity kit used in our study, which has a detection range of 5.00 to 320 ng/mL and a sensitivity of 3.00 ng/mL. Although the lower cut-off limit of the manufacturer-supplied human reference interval for TAFI (1.7–20.0 μg/mL) is lower than the generally accepted human lower cut-off, the highest baboon value obtained (66.3 ng/mL) was still orders of magnitude below human ranges. This may indicate truly lower levels in the Chacma baboon, or more likely (similar to the plasminogen data), that the particular TAFI kit used may not be suitable for quantification of this protein in *Papio ursinus* plasma, as it was designed for use in humans.

Importantly, this again suggests possible structural differences between Chacma baboon TAFI and its human equivalent. However, the inability of a human ELISA to detect baboon protein is not conclusive evidence of a specific structural difference in that protein, especially when considered in isolation. Functional differences may or may not be present and would need to be explored. To better investigate this hypothesis, TAFI activity assays as described by Binette et al. in their study of TAFI activation during sepsis in Yellow baboons (*Papio cynocephalus*) and Olive baboons (*Papio anubis*) [43] may be useful. However, should Chacma baboon TAFI activities also be confirmed to be lower than those of humans, this may have significant implications for assumptions on the level of homology between the fibrinolytic systems of the Chacma baboon and man. Even its homology with other baboons may be diverse, since Yellow and Olive baboons activated TAFI (TAFIa) have already been shown to exert larger effects on clot lysis than human TAFIa does [43]. Should Chacma baboons’ TAFI levels and activities genuinely be very low, an absent or markedly reduced TAFI effect on clot lysis may partly explain Chacma baboons’ short clot lysis times, as TAFI appears to be critical for clot lysis resistance in baboons. TAFIa maximally prolongs clot lysis by approximately 10-fold in clots formed from Yellow and Olive baboon plasma [43] but only results in a 3-fold prolongation in clots formed with human plasma [44].

Significant interspecies differences have been demonstrated between bovine and human TAFI gene sequences [45] and between human, rat, and murine TAFI gene sequences [46]. Bovine TAFI is 77% sequence identical and 86% sequence similar to human TAFI, with sequence comparisons suggesting that bovine TAFI is more stable than the human form [45]. Rat and murine TAFI are reported to be 83% and 84% sequence identical, respectively, to human TAFI. Functional differences have also been shown between rat, murine, and human TAFI [47]. Although the sequence of the Chacma baboon TAFI gene has not been formally studied and compared with human TAFI, it is possible that significant differences also exist across these two primate species, creating a potential future research opportunity.

**Lower α2-antiplasmin concentrations—implications for therapeutic manipulation of the Chacma baboon fibrinolytic system**

Although α2-antiplasmin concentrations/activities were not assessed in our pilot study [9], nor in any of the other preclinical studies in the Chacma baboon aTTP model [26,27,28,29], the substantially lower α2-antiplasmin concentrations found in our present study are consistent with results reported by Collen et al. for an unspecified baboon species, used to investigate the differing susceptibilities to activation by streptokinase and recombinant staphylokinase of the fibrinolytic systems of five representative mammalian species. In their experiments, α2-antiplasmin was present in baboon plasma at concentrations less than half that of human plasma, with α2-antiplasmin purification yielding concentrations of 8 μg/mL in baboon plasma, but 17 μg/mL in human plasma. Although baboon α2-antiplasmin was able to rapidly inhibit baboon plasmin, it did so at a rate >10 times slower than that of human α2-antiplasmin in a human system. Although α2-antiplasmin activities or inactivation rates were not measured in our present study, it is plausible that the lower α2-antiplasmin concentrations can also translate into lower rates of plasmin inactivation, similar to the previously reported findings [18].

This has important potential implications for the therapeutic manipulation of the Chacma baboon fibrinolytic system. Firstly, this may (at least partly) explain our observation that tPA is able to induce greater plasmin generation and shorter clot lysis times in Chacma baboon plasma than in pooled normal human plasma. Secondly, conclusions on the effects of the thrombolytic drugs in any future in vivo aTTP model experiments should be tempered with the knowledge that α2-antiplasmin-mediated plasmin inhibition may be much more profound in humans than in Chacma baboons, leading to a lesser in vivo plasmin effect on human VWF multimers, for instance, than would be possible in the Chacma baboon. Whether the lower α2-antiplasmin levels in Chacma baboons are offset by higher α2-macroglobulin concentrations/activities is unknown and should be investigated in a future study.

**Potential plasmin activity available for therapeutic utilisation**

Regardless of whether Chacma baboon plasminogen may be structurally or even functionally different from human plasminogen as discussed above, it is the overall plasmin activity that can potentially be therapeutically harnessed by the application of various drugs that is ultimately of importance in the future use of thrombolytic drugs in this species in general, and in the aTTP model in particular. It is clear from our current data that streptokinase does not result in meaningful in vitro plasmin activity, even across a range of concentrations, confirming our pilot study findings that streptokinase is not the ideal thrombolytic drug to test the hypothesis that manipulation of the fibrinolytic system may have therapeutic utility in aTTP in this model [9].

Although there is no other published data on Chacma baboon streptokinase-induced plasmin activity, experiments in Hamadryas baboons [14,17] and other unspecified baboon species [13,18] have yielded similar results, with low or absent resultant plasmin activity. However, this observation is not universal, as Collen et al. were able to show that streptokinase induces dose-dependent clot lysis of jugular vein thrombi in Hamadryas baboons [14]. This suggests at least some meaningful streptokinase-induced plasminogen activation but also hints at possible interspecies differences within baboons. Although the presence of anti-streptokinase antibodies (which may have influenced the in vitro response to streptokinase) was not tested for due to cost constraints, anti-streptokinase antibodies as a sole explanation for the virtually non-existent plasmin activity seems unlikely, since all forty baboons had almost identical poor in vitro responses to the drug. Moreover, none of the animals had been previously exposed to streptokinase, and an earlier *Papio hamadryas* study [14] confirmed high antibody titres to only emerge after several weeks of repeated streptokinase treatment.

On the other hand, tPA led to clear activation of *Papio ursinus* plasminogen in our study, to activity levels higher than can be obtained in human plasma using the same amount of activator. Moreover, a tPA concentration of 1800 μg/mL (representing ~67% of the baseline concentration of 2700 μg/mL) rendered approximately the same amount of plasmin activity in baboon plasma as the 2700 μg/mL concentration did in human plasma. Whether this is simply due to lower concentrations in Chacma baboon plasma of plasmin’s principal inhibitor α2-antiplasmin is unclear.

In addition, escalating tPA concentrations led to clear dose-dependent activation of *Papio ursinus* plasminogen, reaching a plateau at either 5400 μg/mL (*n* = 9) or 7200 μg/mL (*n* = 31). This ability to generate convincing plasminogen activation in Chacma baboon plasma in vitro mirrored the in vivo experience of Franz et al. in the Chacma baboon [11]. As also outlined in a later review [12], intravenous infusion of a recombinant human tPA (alteplase; Actilyse^®^) identical to that used in our present study resulted in significant fibrinolytic responses (as measured with rotational thromboelastography) to incremental tPA dosage, with 0.25 mg/kg being the lowest dose at which an identifiable response was noted. Increasing fibrinolytic activity could be appreciated at doses of 0.5 and 1 mg/kg, which was the maximum dose studied. Assuming a total blood volume of 70 mL/kg [48] and a maximum possible haematocrit of 0.49 L/L [49], these doses would correspond roughly to concentrations of 7, 14, and 28 μg/mL plasma, respectively. Interestingly, the lowest concentration used in our in vitro experiments (28.125 μg/mL, which would correspond roughly to an in vivo dose of 1 mg/kg) was only able to induce low levels of plasminogen activation in vitro. However, when viewed in the context of the data from Franz et al. [11], this may be sufficient to generate enough plasminogen activation in the Chacma baboon in vivo to be able to evaluate the effects of tPA in the aTTP model successfully.

It would be prudent to also correlate the maximum future in vivo doses in the aTTP model (and even other experiments) with the current in vitro activity data presented here. The maximum dose should probably not exceed 257 mg/kg, corresponding to the maximum in vitro concentration of 7200 μg/mL used in the present study. However, such megadoses, as were evaluated for streptokinase in our pilot study [9], will probably not be required. Since our data indicate that significant plasmin activity is present even at low concentrations, and early murine experiments with streptokinase showed that a dose representing 20% of the loading dose commonly used in humans to treat PE and stroke was able to successfully resolve aTTP [50], a starting dose in the Chacma baboon aTTP model of as low as 0.3 mg/kg would be rational, as this corresponds to approximately 20% of the human tPA dose per kilogram body weight (100 mg in patients >67 kg body weight, i.e., a maximum dose of approximately 1.5 mg/kg). However, given the uncertainties involved in extrapolating an in vitro plasma concentration directly to an in vivo systemic dose, any proposed dosing strategy remains speculative and exploratory, and should be approached with caution.

Our finding that human recombinant tPA is a more potent activator of Chacma baboon plasminogen than of human plasminogen is consistent with observations reported for the Olive baboon [51]. Using recombinant human tPA as a plasminogen activator as part of a previously described plasmin generation assay [52] with a similar plasmin substrate and method principle to our plasmin activity assay, Tarandovskiy et al. demonstrated a final mean plasmin peak in the ten Olive baboons studied that is approximately 20% higher than the corresponding human value [51]. The difference in plasmin generation rates is even more dramatic, with Olive baboon plasmin generation rates 160% higher than human plasma. These data correlate broadly with our mean baseline Chacma baboon plasmin activity level of 178% (of human activity) and suggest that the plasminogen molecules of baboons, as a broader group, may be more susceptible to activation by human recombinant tPA than human plasminogen is.

Interestingly, although Rhesus monkeys had plasmin generation rates more similar to human rates than Olive baboons had, their final mean plasmin peak was even higher than those of Olive baboons, suggesting that baboons may be a more suitable fibrinolysis model than Rhesus macaques. These authors caution that even within the most similar species (baboons, Rhesus macaques and humans), fibrinolytic system differences can be detected [51], which could potentially also be applicable across different baboon species as well.

The greater plasmin activity generated by recombinant human tPA in baboon plasma compared with human plasma has several obvious implications for the use of recombinant tPA in the Chacma baboon aTTP model in terms of translatability. Firstly, any possible utility of the drug in the treatment of aTTP in humans may be overestimated since the same level of plasminogen activation (and the resultant possible effect on VWF multimer composition or GPIb interaction) achieved in the Chacma baboon aTTP model in vivo may not be attained in vivo in humans. Secondly, a different dosage may be required in vivo in humans than suggested by any future Chacma baboon model data. Should this drug be successful in treating aTTP in the Chacma baboon model, it is possible that a different dose will be required in humans to achieve the same effect. Thirdly, the more potent plasmin activity has safety implications. Since these animals may now have a higher bleeding risk than expected with a comparable dose in vivo in humans, any excessive bleeding in future experiments should be interpreted with caution and not summarily equated to a similarly increased risk in humans.

**Actual clot lysis ability of tPA-activated Chacma baboon plasmin**

The plasmin activity generated by tPA also resulted in actual clot lysis in vitro and not merely in plasmin activity measurable by chromogenic assay. This is an important in vitro validation of the effects of tPA before proceeding to any future in vivo study in this model. The greater plasmin activity generated by tPA in Chacma baboon plasma than in human plasma also appears to translate into slightly quicker clot lysis times in vitro. This also confirms the implications for its use in the Chacma baboon aTTP model in terms of efficacy, dosage, and safety, already outlined above. Interestingly, in the only other study to assess the clot lysis ability of the Chacma baboon [6], clots were found to be more resistant to fibrinolysis than human clots, although this study assessed fibrinolysis using thromboelastometry, and plasmin generation was not actively induced using a thrombolytic drug such as tPA. However, this does highlight the lytic potency of tPA in Chacma baboon plasma, given the apparent intrinsic resistance of Chacma baboon clots to fibrinolysis. In contrast, Cynomolgus monkeys (*Macaca fascicularis*) and humans are reported to have similar Maximum Lysis percentages on thromboelastometry [53], again highlighting the dangers of assuming uniform clot lysis abilities across all primate species.

In a study of thrombin-thrombomodulin’s activation of TAFI in a Yellow baboon and Olive baboon *Escherichia coli*–induced sepsis model, Binette and co-workers performed clot lysis time assays using a method similar (although not identical) to ours [43]. Interestingly, they found that when activated baboon TAFI was neutralised by adding potato tuber carboxypeptidase inhibitor (PTCI), clot lysis times were 89% lower in the PTCI-exposed plasma than in controls, suggesting a significant role for activated baboon TAFI in rendering clots resistant to tPA-induced fibrinolysis. The very low levels of TAFI in Chacma baboon plasma (if accurate) may therefore also possibly contribute to quicker clot lysis compared with human plasma, which, in combination with the Chacma baboon’s propensity for greater tPA-induced plasminogen activation, culminates in an overall increased susceptibility to tPA-induced fibrinolysis than would be achieved in humans. Moreover, lower Chacma baboon TAFI concentrations may be particularly important in this context, as it appears that baboon TAFIa may have a larger effect on clot lysis than human TAFIa does. TAFIa was shown to maximally prolong Yellow baboon and Olive baboon clot lysis by a factor of almost 10 [43]. In contrast, only a 3-fold prolongation was observed in clots formed with human plasma [44].

**Effects of ABO blood group and sex**

Fascinatingly, in contrast to a previous study in South Africa of ABO blood groups in two Chacma baboon subspecies (*Papio ursinus orientalis* and *P.u. occidentalis*), where no baboon was found to be of blood group O in either of the two subspecies [54], the majority (*n* = 30; 75%) of animals in our study were of blood group O. No baboons in our study typed as group B or AB either, while other studies reported the distinct presence of the B allele, similar to humans [54,55]. This may be a consequence of the fact that only purpose-bred baboons from the UFS Animal Research Centre’s breeding colony were used for this study without the addition of any wild-caught animals, which may have limited the genetic diversity of the group. A vital novel finding which has implications for any future experiment involving haemostasis in this species is the significantly higher fibrinogen levels (*p* = 0.0355, Hedges’ g = 0.7914, 95% CI: 0.0689–1.514) found in blood group O baboons, although the exploratory nature of these analyses, as well as the small subgroup sizes, should be acknowledged. Since fibrinogen concentrations are reported not to be affected by ABO blood groups in various human populations [56,57], this is an essential interspecies difference that should be taken into consideration when planning future Chacma baboon model experiments.

In addition, in contrast to humans, where no sex-related difference is evident [58,59,60], fibrinogen concentrations were significantly lower in male baboons (*p* = 0.0134, Hedges’ g = 0.7522, 95% CI: 0.0944–1.4100), so that female baboons seem “primed to clot”. This has implications for the selection of individual test animals for any future experiments in this species and the sex-based composition of any larger groups and subgroups, which, depending on the experimental design, should now arguably include both male and female animals. This recommendation is strengthened by recent evidence that megakaryocyte biology and prothrombotic transcriptional programmes, for example, can differ according to sex [61].

Similar to humans [62], male baboons had significantly higher PAP complex concentrations (*p* = 0.0188, Hedges’ g = 0.8252, 95% CI: 0.1632–1.4872), which possibly indicate higher baseline background fibrinolytic activity than females, although there is no supporting evidence for this from the plasmin activity or modified clot lysis time data. Any sex-related differences apparent from our ultimately exploratory data should be interpreted within the context of the small subgroup sizes and require further validation.

A summary of all the novel provisional reference intervals calculated from our data is provided in Table 2 below for those parameters where meaningful results could be generated, i.e., where assays were not suspected of being affected by significant interspecies differences (e.g., TAFI). One of the major strengths of these provisional reference intervals compared with other studies (and indeed of this entire study) is the large number of animals (*n* = 40) included in the calculations. Apart from two earlier studies conducted by our group on this same cohort of 40 Chacma baboons, which investigated their thrombin generation capabilities [63] and natural anticoagulants and factor VIII levels [64], other studies which focused specifically on establishing reference intervals for other haemostatic parameters such as platelet function and thromboelastometry in the Chacma baboon included only 24 [65] or 25 [6] animals, respectively. Moreover, unlike our studies, both these studies only included male baboons, which may bias the results. Nevertheless, in accordance with the Clinical and Laboratory Standards Institute (CLSI) guidelines for the establishment and verification of reference intervals [66], ABO-specific and sex-specific reference intervals were not calculated using our data due to the limited numbers of animals per subgroup, even for those parameters where differences were apparent. The lack of laboratory assay standardisation for many of these analytes is another important limitation to consider when comparing our data with those of previous or future studies, especially when discrepant results are obtained. At least some of these differences could be attributed to the use of assays from different manufacturers.

The interindividual variation, represented by the CV, is also compared in Table 2 with a selection of published human interindividual (between-subject; CV_g_) biological variation values, where available. Many fibrinolytic system components displayed interindividual variation comparable to that of humans. This is encouraging in terms of the continued suitability of this species as an animal model of human haemostatic disease and the translatability of results.

Fibrinogen and especially α2-antiplasmin showed greater variability than in humans, and results from future experiments in this species should be interpreted with caution and with this higher interindividual variability in mind. The Chacma baboon’s extremely high interindividual CV for α2-antiplasmin (270.5%) is somewhat unexpected since both interindividual (CV_g_) and intraindividual (CV_i_) human biological variation are reported to be well below 10%, at 7.1% [71] and 6.6% [73,74], respectively. Intraindividual variation was not assessed in the present study but may be the subject of future research.

Chacma baboon interindividual variation for tPA was lower than human interindividual variation. This could be explained by the sampling times of the specimens. Human tPA levels display a prominent circadian rhythm, characterised by low early morning levels and high afternoon peaks, resulting in a peak-to-trough difference of almost 150% [75]. Since all baboon specimens were collected at roughly the same time of day (mid-morning), the effect of such a circadian rhythm (if also present in Chacma baboons) would have been negated, leading to less interindividual variation than reported in humans.

**Future studies**

Perhaps the most pertinent opportunities lie in the sequencing of the genes of proteins found to have such discrepant results with human reference intervals to suggest fundamentally different structures: plasminogen and TAFI. Investigating the level of homology of these genes between the various commonly used baboon species and humans may also indicate whether any other baboon species may be superior to *Papio ursinus* as a model of human haemostasis.

Several components related to fibrinolysis and the fibrinolytic system also remain to be explored. Reference intervals for factor XIII, PAI-2, α2-macroglobulin, and baseline D-dimer concentrations will all add value to future studies.

Intraindividual variation (as opposed to interindividual variation) was not assessed for any of the parameters in the present study and may be a beneficial future research endeavour. In addition, it would be valuable to know whether Chacma baboons are also subjected to the same diurnal variations reported in humans for parameters such as the tPA concentration. Perhaps the most vital potential future study arising from our data is a possible repeat of our pilot study but using recombinant human tPA as a thrombolytic drug. This should be done using a range of tPA dosages, starting at a low dose of 0.3 mg/kg, to provide proof-of-concept that thrombolytic drugs (perhaps even at very low doses) can be used to treat aTTP in humans. Future studies in this species where fibrinogen concentrations are important should consist of both male and female baboons and animals from O and non-O ABO blood groups.

## 4. Materials and Methods

Ethics statement

Ethics approval was obtained from the University of the Free State (UFS) Animal Research Ethics Committee before commencement of the study (UFS-AED2019/0054). Protocols are evaluated against the South African National Standard for the care and management of laboratory animals (SANS10386:2008) and must comply with the commonly accepted ‘3Rs’ principles. All the methods, where applicable, are reported in agreement with the ARRIVE guidelines (https://arriveguidelines.org).

**Experimental animals, sample acquisition and preparation**

The same cohort of animals on which our group had previously studied the thrombin generation capability [63] and natural anticoagulants and factor VIII levels [64] of the Chacma baboon was used for this study. Samples from the same sampling event as for these previous studies [63,64] were used for the present study. Briefly, as previously described [63], only purpose-bred animals were sampled, and no specimens were collected from other animal species. Routine care and phlebotomy were conducted at the Animal Research Centre of the UFS (Bloemfontein, South Africa). Blood was collected from 51 baboons, from which a subgroup of 40 baboons was selected based on their ABO blood groups and sex, to have as broad a representation of the different ABO blood groups and sexes as possible. As previously reported [63], only 10 baboons were of non-O ABO blood groups and were all included. The rest were all of group O, from which the 30 animals with the largest available number of plasma aliquots (that would be able to accommodate all the planned tests) were selected. Only 14 animals were male and were all included. All 11 baboons not selected for further testing were female and of group O. All 51 animals screened were either adults or young adults.

The exact genetic heterogeneity of the animals is unknown, but it is unlikely that they were all derived from the same original breeding pair (or pairs) since various baboons were historically used for breeding purposes in the colony. The environmental factors were identical for all animals in the colony [63].

As previously reported [63], all blood sampling and administration of sedation were performed by a South African Veterinary Council (SAVC) registered veterinarian. Blood sampling was performed under sedation, with ketamine hydrochloride given intramuscularly every 30 min at 1 mg/kg body weight, as needed. All doses were based on the measured body weight of each animal. Animals’ eyelid and toe pinch reflexes were monitored during sedation. Measures to prevent hypothermia during sedation were also followed. All blood sampling procedures were performed with strict aseptic technique.

Blood specimens were collected into BD Vacutainer^®^ tubes (BD Biosciences, Franklin Lakes, NJ, USA) and were drawn from a 20-gauge IV catheter inserted in the femoral/cephalic/great saphenous vein by an experienced veterinarian. A maximum of fifteen separate 5 mL specimen tubes, each containing 0.5 mL 3.2% sodium citrate, were filled with 4.5 mL blood per tube, for a maximum total of 67.5 mL citrated whole blood per baboon. Sampling volumes, therefore, did not exceed 10% of the total blood volume, based on a total blood volume of 70 mL/kg measured body weight [48]. All animals were returned post-sampling to the colony without any adverse events [63].

Platelet-poor plasma was prepared by centrifugation of whole blood at 1500× *g* for 15 min at room temperature, then aliquoted and stored at −80 °C within 90 min of specimen collection [63].

**Assays**

**Fibrinogen**

Fibrinogen was measured using the Clauss fibrinogen method, in an ISO 15189-accredited (Geneva, Switzerland, 2012) laboratory, on a Sysmex CS-2100i^®^ fully automated coagulation analyser (Sysmex, Kobe, Japan, supported locally by Siemens), according to the manufacturer’s instructions. Briefly, in principle, an excess of the enzyme thrombin is added to test plasma, which converts the soluble plasma protein fibrinogen into its insoluble polymer, fibrin. The clotting time for the diluted plasma is inversely proportional to the fibrinogen concentration in the test plasma [76].

**Plasminogen**

A Glu-Plasminogen ELISA kit (TECHNOZYM^®^ Glu-Plasminogen ELISA kit, Technoclone GmbH, Vienna, Austria) was used to measure Glu-plasminogen levels. Glu-plasminogen is the predominant native plasminogen that is converted to active plasmin through two consecutive cleavage actions by specific plasma proteases [77]. Plasmin itself also modifies Glu-plasminogen and exposes lysine binding sites to form the Lys-plasminogen variant that, through its superior fibrin binding and activation capacity, mediates a positive feedback mechanism leading to the enhanced conversion of plasminogen to plasmin [77]. By assessing Glu-plasminogen instead of Lys-plasminogen or plasmin, the total potential initial pool available for plasmin formation is therefore considered.

The Glu-Plasminogen ELISA test is a chromogenic solid-phase enzyme immunoassay, and the mAbs employed in this test system recognise only uncleaved Glu-plasminogen. The results are, therefore, not affected by the presence of PAP complexes or plasmin-modified Lys-plasminogen.

The ELISA test strips, which had been pre-coated with a monoclonal anti-plasminogen antibody and blocked with 1% bovine serum albumin, were incubated with the test samples and controls. A POX-conjugated monoclonal anti-plasminogen antibody was added, followed by the TMB substrate. Measurements were performed using an ELISA reader with a 450 nm filter, and concentrations were read from the calibration reference curve.

**tPA**

tPA levels were measured using a tPA antigen ELISA kit (TECHNOZYM^®^ t-PA Antigen ELISA kit, Technoclone GmbH, Vienna, Austria) according to the manufacturer’s instructions. The kit consisted of ELISA test strips pre-coated with an anti-tPA monoclonal capture antibody. After incubation of the test plasma, a POX-conjugated anti-tPA antibody was added, followed by the chromogen TMB as a substrate. Measurement was performed using an ELISA reader at 450 nm, and concentrations were read from the calibration reference curve.

**PAI-1**

PAI-1 levels were also measured using an ELISA method, according to the manufacturer’s instructions. The PAI-1 antigen ELISA kit (TECHNOZYM^®^ PAI-1 Antigen ELISA kit, Technoclone GmbH, Vienna, Austria) consisted of ELISA test strips coated with anti-PAI-1 monoclonal capture antibody, to which test plasma was added, followed by a POX-conjugated anti-PAI-1 antibody. Upon addition of the TMB chromogenic substrate, a colour change ensued, which is directly proportional to the amount of PAI-1 in the test plasma. This colour change was measured at 450 nm using an ELISA reader and compared with a calibration reference curve.

**PAP complex**

PAP complex levels were also measured using an ELISA kit (TECHNOZYM^®^ PAP Complex ELISA Kit, Technoclone GmbH, Vienna, Austria) according to the manufacturer’s instructions. The PAP complex ELISA kit consisted of ELISA test strips pre-coated with a monoclonal capture antibody directed against the neoantigen in the PAP complex, in which test plasma and controls were incubated. A conjugated, polyclonal anti-plasminogen antibody (POX-conjugated antibody) was added, followed by its TMB substrate. An ELISA reader with a 450 nm filter was used to perform the measurement, and concentrations were read from the calibration reference curve.

**TAFI**

TAFI concentrations were determined using a high-sensitivity ELISA kit (Human TAFI ELISA Kit, Elabscience, Houston, Texas, USA) which uses the Sandwich-ELISA principle, according to the manufacturer’s instructions. The micro-ELISA plate provided in the kit had been pre-coated with an anti-human TAFI-specific capture antibody. Standards or samples were added to the micro-ELISA plate wells and combined with the specific antibody. Then a biotinylated detection antibody specific for human TAFI and an Avidin-HRP conjugate were added successively to each microplate well and incubated. Free components were washed away, and the substrate solution was added to each well. The enzyme-substrate reaction was terminated by adding a stop solution, and the optical density (OD) was measured spectrophotometrically at a wavelength of 450 nm ± 2 nm. The OD value is proportional to the concentration of TAFI in the sample, which was then calculated by comparing the OD of the sample to the standard reference curve.

ELISA kits with differing sensitivities and quantification ranges are available for the measurement of TAFI concentrations. We selected a high-sensitivity kit, with a detection range of 5.00 to 320 ng/mL and a sensitivity of 3.00 ng/mL. Although human reference intervals for TAFI are usually in the μg/mL range, this kit was selected to be certain that low concentrations of TAFI would still be detected and accurately quantified, even if present at exceedingly low levels. If this kit demonstrated baboon sample TAFI values to be >320 ng/mL, a second kit with a higher detection range would have been used.

**α2-antiplasmin**

α2-antiplasmin levels were determined using the SimpleStep ELISA^®^ kit (Abcam, Cambridge, United Kingdom), which employs an affinity tag labelled capture antibody and a reporter-conjugated detector antibody, for immunocapture of the sample analyte of interest. This complex (capture antibody/analyte/detector antibody) is consequently immobilised by an anti-tag antibody, coating micro-ELISA plate wells. Samples or standards were added to the wells, followed by the antibody mixture. After incubation at room temperature for 1 h, the wells were washed to remove any unbound substances. TMB development solution was added and catalysed by HRP during incubation, leading to blue colouration. The reaction was then stopped by adding the stop solution, which completes the colour change from blue to yellow. The resultant OD is proportional to the amount of bound α2-antiplasmin and was measured spectrophotometrically at 450 nm. The final concentration of α2-antiplasmin in each sample was then calculated by comparing the OD of the sample to the standard reference curve.

**Plasmin activity**

Baseline streptokinase-induced plasmin activity was measured chromogenically, using a method obtained from the IRF Life Sciences Laboratory for Thrombosis Research at the Kulak Kortrijk Campus of KU Leuven, Kortrijk, Belgium (C Tersteeg 2017, personal communication, 30 August). Briefly, a commercially available chromogenic plasmin substrate *N*-Tosylglycyl-L-prolyl-L-lysine 4-nitroanilide acetate salt (TOS-Gly-Pro-Lys-4-NA; Merck KGaA, Darmstadt, Germany) was added to 2 μL buffered test plasma, after which streptokinase (10 μL of a 400 IU/mL solution) was added, and plasmin activity measured kinetically at 405 nm for 2 h with an ELISA reader set at >37 °C. Readings were compared with a standard curve derived from commercially available pooled buffered normal human plasma and reported as a percentage. This procedure was also performed using a comparable amount of recombinant human tPA (alteplase; Actilyse^®^, Boehringer Ingelheim, Ingelheim am Rhein, Germany) as plasminogen activator (10 μL of a 2700 μg/mL solution). The total doses of streptokinase and alteplase used to treat STEMI in humans are 1,500,000 IU and 100 mg (in patients >67 kg), respectively [78]. Therefore, the concentrations of these solutions represent approximately 0.03% of the total STEMI treatment dose per millilitre.

These procedures were repeated using escalating pharmacological-level (and not physiological-level) concentrations of streptokinase and tPA as activators, until the point of maximum streptokinase-generated plasmin activity and maximum tPA-generated plasmin activity was reached. This approach was followed in order to ascertain the absolute maximum plasmin activity potentially attainable with each activator. It was done by adding 10 μL of a streptokinase or tPA solution at the following concentrations until a plateau in plasmin activity was observed:

- Streptokinase: 800 IU/mL, 3200 IU/mL, 25,600 IU/mL
- tPA: 28.125 μg/mL, 56.25 μg/mL, 112.50 μg/mL, 225 μg/mL, 450 μg/mL, 900 μg/mL, 1800 μg/mL, 2700 μg/mL, 3600 μg/mL, 5400 μg/mL, 7200 μg/mL.

**Modified clot lysis time**

Plasma clot lysis was assessed based on a modification of the standardised method of clot lysis time (CLT) determination proposed by Pieters et al. for the Subcommittee on Factor XIII and Fibrinogen, and the Subcommittee on Fibrinolysis, of the Scientific and Standardization Committee (SSC) of the International Society on Thrombosis and Haemostasis (ISTH) [79]. Briefly, buffered plasma with and without tPA is clotted at 37 °C with an activation mix consisting of human α-thrombin, CaCl_2_ and buffer, in the presence of an excess of phospholipids, in a 96-well ELISA plate. Changes in turbidity are then read with an ELISA reader, measuring the absorbance at 405 nm every 10 s up to 30 min (180 readings) and then every minute until the clots have broken down or up to 180 min (*n* = 150 readings). The timepoint at which 50% clot lysis has occurred is defined as the CLT. This method was used as a basis for the modified CLT, minreconfigured for baboon plasma (please see modifications below).

The 50% CLT was calculated from a graph in the Prism software programme (version 11), using a Boltzmann sigmoidal curve fitting. The 50% CLT was calculated as the time in minutes from the midpoint of the ascending curve (from clear to maximum turbidity) to the midpoint in the descending curve (the transition from maximum turbidity to the final baseline turbidity). These midpoints were identified by applying two separate sigmoidal curve fittings to the ascending and descending parts of the turbidity curves. The Prism software programme was used to calculate the midpoints from the fitted curves. The time of the midpoint of the ascending curve was then subtracted from the time of the midpoint of the descending curve to provide the 50% CLT.

According to the original method, each laboratory should first determine the tPA concentration to be used locally by establishing the concentration at which a CLT of 100 ± 5 min is obtained on lyophilized NIBSC plasma. It is suggested to start these experiments with tPA concentrations in the range of 20–40 ng/mL. It must be emphasised that these tPA concentrations represent more *physiological* levels of tPA, in the ng/mL range (the human reference interval for tPA is 2–8 ng/mL), with which to assess physiological clot lysis (while still shortening clot lysis times to practically feasible and measurable times in vitro). These concentrations should not be directly compared with the μg/mL *pharmacological*-level concentrations of tPA defined above to establish the absolute maximum plasmin generation potential of tPA. The clot lysis assay is intended to offer a more physiological assessment of the test plasma’s inherent clot lysis ability under physiological circumstances (rather than to provide an assessment of maximum clot lysis ability) while still providing practical lysis times, measurable in the laboratory.

The procedure was modified as follows:

- To apply the assay to the individual baboon plasma samples, a tPA concentration of 225 ng/mL was selected, since this was the lowest concentration able to elicit complete clot lysis in both pooled normal human plasma and pooled baboon plasma samples, but resulted in a CLT of ±10 min on pooled baboon plasma and ±13 min on pooled normal human plasma. The pooled normal human plasma used for this experiment was prepared from 3.2% citrated plasma previously collected before 11:00 in the morning from 20 fasting, healthy individuals. Exclusion criteria were pregnancy, elevated temperature, known blood disorders, use of oral contraceptives, or use of acute or chronic medication. Whole blood specimens were kept at room temperature after collection for no longer than 3min0 min and then centrifuged at 2000× *g* for 20 min to prepare platelet-poor plasma. Plasma of all individuals was pooled, mixed and aliquoted before freezing at −70 °C. Pooled baboon plasma was prepared by pooling and mixing previously prepared individual baboon plasma aliquots that had been thawed in a water bath at 37 °C just before use.
- All plasma samples were clotted with locally available bovine thrombin (Dade^®^ Thrombin, Siemens Healthcare GmbH, Erlangen, Germany) at a concentration of 2.5 NIH U/mL in the final activation mixture instead of human α-thrombin, due to ease of availability and cost constraints.
- The commercial phospholipid preparation recommended by Pieters et al. (Rossix, Cat no: PL604T, 0.5 mmol/L) was not used due to cost constraints. Instead, phospholipids were prepared locally as follows: 50 mL human platelet-rich plasma from the South African National Blood Service was washed twice with Tris-buffered saline and frozen for 20 min at −80 °C, thawed at 37 °C, and frozen again. This freeze–thaw cycle was repeated until the platelet count was <20 × 10^9^/L, confirming that virtually all platelets were lysed, yielding a platelet lysate suspension of phospholipids.

**ABO blood group**

ABO blood grouping was based on the principle of antibody-induced erythrocyte clumping. Erythrocytes with a given ABO blood group antigen on their surface will agglutinate when exposed to antibodies directed against the given antigen. Briefly, 2–5% erythrocyte suspensions were prepared and mixed with anti-A, anti-B, and anti-AB antibodies to determine the red cell ABO phenotype (forward grouping). Additionally, in the ABO blood group system, as an individual’s serum will naturally contain antibodies directed against the antigen(s) lacking on their erythrocytes, reverse grouping was performed as a confirmatory step by adding test serum to red cells of known ABO blood group.

**Statistical analysis**

Statistical analysis was performed using the EP Evaluator^®^ (version 10.3.0.556, Data Innovations, 2013), Microsoft Excel^®^, and XLSTAT-Biomed^®^ statistical analysis software systems. Quantitative data were summarised using descriptive statistics, namely frequencies and percentages for categorical data, and means, medians, standard deviations (SDs), coefficients of variation (CVs), and percentiles, for continuous data.

Biological reference intervals were calculated for all relevant parameters, following the CLSI guidelines [66]. The central 95% interval of the distribution, i.e., the interval from the 2.5th percentile to the 97.5th percentile (or upper limit of this interval if appropriate), was defined as the biological reference interval/limit, as stipulated in ISO standard 15189 [80]. The CVs calculated were also compared with published human biological variation values.

To assess whether the distribution of the data was normal, D’Agostino-Pearson tests were performed on datasets with repeated values, and Shapiro–Wilk tests on all other datasets.

An unpaired Student’s *t*-test was performed to determine if there was a significant difference (*p* < 0.05) between ABO blood group O and non-O baboons and between male and female baboons for all parameters.

## 5. Conclusions

Fibrinogen, along with most role players of the fibrinolytic system, has ranges and characteristics comparable to its human equivalents. Plasminogen and TAFI, however, likely have structural/functional differences which can affect the assay values obtained and can also impact the treatment of aTTP with thrombolytic drugs in this model. While streptokinase cannot be used, recombinant human tPA may be an option. Critically, regardless of the reason for its greater plasminogen activation in the Chacma baboon, recombinant human tPA induces sufficient plasmin activity in *Papio ursinus* plasma to lead to brisk clot lysis. Since significant plasmin activity was present even at low tPA concentrations and considering that a streptokinase dose equivalent to 20% of the human PE treatment dose was effective in resolving aTTP in mice [50], a low tPA dose of 0.3 mg/kg followed by escalating doses (up to 257 mg/kg if needed) could probably be attempted in the Chacma baboon aTTP model in vivo.

Except for plasminogen and TAFI, the rest of the components of the fibrinolytic system of the Chacma baboon appear broadly similar to their human counterparts, enabling the continued use of this species as a model of human haemostasis, provided quantitative results are interpreted within the context of the novel provisional reference intervals reported above and the limitations and interspecies differences outlined above are considered. The most important of these limitations and differences would be the findings that streptokinase does not activate the Chacma baboon fibrinolytic system but that recombinant human tPA elicits even greater plasminogen activation in *Papio ursinus* than it does in pooled normal human plasma under the present assay conditions, that sex-related and ABO-blood-group-related differences are imperative when measuring fibrinogen in this species, that assays designed to measure human plasminogen and TAFI may not be usable in this species, and that fibrinogen and especially α2-antiplasmin are subject to considerable interindividual biological variation in these animals.

## Figures and Tables

**Table 1:** The range, median, mean, standard deviation (SD), coefficient of variation (CV), and central 95% interval of fibrinogen, plasminogen, tissue-type plasminogen activator (tPA), plasminogen activator inhibitor-1 (PAI-1), plasmin-α2-antiplasmin (PAP) complex, thrombin activatable fibrinolysis inhibitor (TAFI), and α2-antiplasmin values.

Parameter, with Human Reference Range	Range	Median	Mean	SD	CV%	Central 95% Interval
Fibrinogen 1.70–4.20 g/L [19]	1.16–4.26	1.83	2.02	0.68	33.67	1.28–4.00
Plasminogen 60–250 μg/mL [20]	1.5–28.2	3.1	2.4	4.1	132.0	1.8–4.2
tPA 2–8 ng/mL [21]	1.4–4.0	1.9	2.0	0.5	24.9	1.6–3.0
PAI-1 7–43 ng/mL [22]	1.9–32.3	9.6	11.3	7.1	63.1	2.6–30.1
PAP complex 0–514 ng/mL [23]	99.9–340.8	130.8	141.8	40.8	28.8	109.0–215.8
TAFI ng/mL 1700–20,000 ng/mL [24]	0.0–66.3	1.8	9.7	15.4	158.8	0.0–59.3
α2-antiplasmin 25–50 μg/mL [25]	1.2–95.8	1.5	5.7	15.3	270.5	1.2–21.8

**Table 2:** Consolidated provisional reference intervals and interindividual variation in the Chacma baboon (*n* = 40) of fibrinogen and the various ultimately testable quantitative parameters of the fibrinolytic system, compared with the local human reference intervals and published human interindividual biological variation, respectively, where available.

Parameter	Chacma Baboon Reference Interval (2.5–97.5%)	Human Reference Interval for Assay	%CV	Human %CVg, with Reference
Fibrinogen (g/L)	1.28–4.00	1.70–4.20	***33.67***	17.0 [67] 20.2 [68] 21.8 [60]
tPA (ng/mL)	1.6–3.0	2–8	***24.9***	47.8 [69] 45.6 [68]
PAI-1 (ng/mL)	2.6–30.1	7–43	63.1	70.9 [68] 91 * [70]
PAP complex (ng/mL)	109.0–215.8	0–514	28.8	26.0 [71] 29.3 [68]
α2-antiplasmin (μg/mL)	1.2–21.8	25–50	***270.5***	7.1 [71]
tPA-induced plasmin activity (%)	124–261	±100	26	N/A
tPA-induced modified clot lysis time (minutes)	8.5–12.6	±13.4	11.6	10.2 [72]

%CV = Coefficient of Variation; %CVg = Interindividual (between-subject) biological variation; N/A = Not Available. * Postmenopausal women. %CVs that differ by >10 from the closest human %CV_g_ are shown in bold italics.

## Data Availability

The original contributions presented in this study are included in the article. Further inquiries can be directed to the corresponding author. The datasets used and/or analysed during the current study are available from the corresponding author on reasonable request.

## Abbreviations

The following abbreviations are used in this manuscript:

ADAMTS13	A disintegrin and metalloproteinase with a thrombospondin type I motif, member 13
aTTP	Acquired thrombotic thrombocytopenic purpura
CLSI	Clinical and Laboratory Standards Institute
CLT	Clot lysis time
CV	Coefficient of variation
DIC	Disseminated intravascular coagulation
ELISA	Enzyme-linked immunosorbent assay
PAI-1	Plasminogen activator inhibitor-1
PAP	Plasmin-α2-antiplasmin
POX	Peroxidase
SD	Standard deviation
TAFI	Thrombin activatable fibrinolysis inhibitor
TAFIa	Activated thrombin activatable fibrinolysis inhibitor
tPA	Tissue-type plasminogen activator
VWF	Von Willebrand factor
